# Regional assessment of human-caused ecological risk in the Poyang Lake Eco-economic Zone using production–living–ecology analysis

**DOI:** 10.1371/journal.pone.0246749

**Published:** 2021-02-08

**Authors:** Hui Wang

**Affiliations:** 1 College of Tourism and Geography, Jiujiang University, Jiujiang, China; 2 College of Geography and Planning, Nanning Normal University, Nanning, China; Gebze Teknik Universitesi, TURKEY

## Abstract

In this study, based on the regional land-use risk space division (regional ecological risk source/receptor space identification) using production–living–ecology analysis, three spatial function indexes, i.e., production, living, and ecology function indexes, were proposed for regional ecological risk assessment (RERA) with respect to human disturbance. The first two indexes can be regarded as regional ecological risk source indexes, whereas the final index can be regarded as a regional ecological risk receptor index. Using an artificial assignment method based on the land-use types and Defense Meteorological Program Operational Line-Scan System (DMSP/OLS) nighttime light intensity data, these three spatial function indexes were effectively manifested. By incorporating these indexes with eco-environmental vulnerability proxies, an RERA framework was established and applied in the Poyang Lake Eco-economic Zone (PLEZ), which is an ecological-protection and economic-development coordination-oriented region in China. The results suggest that (1) the DMSP/OLS nighttime light intensity data correlated well with the spatial distribution of regional urban/town areas; consequently, it was reasonable to use this dataset for representing regional production-living function space (urban/town area). (2) Overall, the forestlands and winter waterbodies of Poyang Lake were in the high-risk grade, and so did the Nanchang City construction land area; in contrast, the final risk levels of winter wetlands and croplands were relatively low. (3) Owing to the highest human disturbance (including both production and consumption human activities) and eco-environmental vulnerability level, urban/town areas such as Nanchang City had the highest final risk grade. (4) The low, medium, high, and very high-risk grades accounted for 21.22%, 39.53%, 36.31%, and 2.94% of the region, respectively. I believe that the proposed land use function indexes will be helpful in conducting human-caused RERA research in the future. Furthermore, the assessment results can provide a scientific basis for regional ecological risk management within the PLEZ.

## 1. Introduction

Besides climate change and natural hazards, human activity has been identified as one of the main sources of regional ecological risk [1–4]. With the ongoing urbanization and industrialization, several issues such as natural resource over-exploitation, ecosystem damage, and environmental pollution are becoming increasingly serious [5]. Therefore, sustainable development that focuses on the coordination and optimization of the relationship between human production-consumption activities and eco-environmental protection is also becoming increasingly popular globally. Consequently, ecological risk management (ERM), which emphasizes on the prevention of ecological destruction and pollution events, and ecological risk assessment (ERA), which provides a scientific decision basis for ERM, have attracted attention from scientists, politicians, and the general public [3, 4]. Related research and practices have proved that ERM and ERA play a crucial role in environmental management and biological/ecological protection, and subsequently, in regional sustainable development [2, 6]. Based on its development process, ERA can be divided into several sub-phases, i.e., the budding stage, human health assessment stage, ERA stage, and regional ERA (RERA) stage [7]. From ERA to RERA, the relevant scale has expanded from single points to regional and landscape scales. With an increasing scale, the focus has shifted to the analysis of spatial heterogeneity of risk sources, receptors, and final integrated risk on a regional scale [1–4]. Consequently, regional ecological risk assessment (RERA) and management (RERM) are becoming important topics in regional eco-environmental management and protection research [4]. A series of methodologies have been developed and practiced in RERA research, such as the “Three-Step Framework” proposed by the United States Environmental Protection Agency [8], Relative Risk Model (RRM) by Landis and Wiegers [9], and the Procedure for Ecological Tiered Assessment of Risk by Moraes et al. [10]. All these provide useful references for related research, such as normally including three basic indexes in the RERA process (i.e., risk source intensity index, risk receptor ecological-value index, and regional eco-environmental vulnerability index), and emphasizing on the spatial differentiation analysis finally [1, 3, 4]. Another important issue in the human-caused RERA study is the identification of ecological risk sources and receptors and the selection of corresponding indexes. Normally, ecological risk describes the potential decline in the ecosystem health level and provision capacity of ecosystem services [11]; therefore, in RERA research, the regional ecosystem services function is often selected as an ecological risk receptor [3–5, 11]. However, by comparison, the identification and selection of regional ecological risk source indexes with respect to human activities are normally difficult and unclear. Thus, from the viewpoint of ecological risk source indexes selection, how to characterize the spatial differentiation features of regional human activities within the human-caused RERA research is of great importance.

In a recent study, the production-living-ecology analysis was adopted to establish a regional ecological risk source-receptor index system within human-caused RERA framework, among which the production and living functions of regional land-use space were recognized as ecological risk source indexes whereas the ecology function of land-use space was identified as an ecological risk receptor index [12]. To the best of my knowledge, this study is the first to incorporate the regional production–living–ecology function analysis into RERA research with respect to human activities. Compared with traditional human-caused RERA studies, such as employing land-use intensity [2, 13, 14] or landscape disturbance index [15, 16] as an ecological risk source proxy, the production–living–ecology division scheme is a new analysis of human effects and ecological risk source-receptor indicator selection system on a regional scale. This will undoubtedly promote the progress in the human-caused RERA studies in the future. Unfortunately, the economic statistics data method used in that study [12] is only able to express spatial differences of production and living functions, i.e., human disturbance level, between different administrative units on the regional scale; in other words, this method is unable to exactly manifest the spatial pattern of intensities of regional production and living functions based on land use/land cover data. Given that the production-living-ecology function division scheme is acquired based on land use function concept, the artificial assignment method [17, 18] based on land-use data could be the more accurate way to characterize spatial heterogeneities of regional production and living functions regionally. Therefore, using the artificial assignment method of regional production–living–ecology function based on land-use data, this research aims to examine the effectiveness of this new method for human-caused RERA purpose.

Land-use space plays an important role in the occurrence and absorption of human-caused ecological risks on a regional scale. It can be used as spatial surrogate for human activities. From the viewpoint of land-use function, regional land-use space can be differentiated into three dominating function types, i.e., production function, living function, and ecology function, in line with their main land function [19–21]. The concept of production–living–ecology function was first proposed in the report of the 18th National Congress of the Communist Party of China [22]. Since then, this land-use function division scheme has attracted considerable attention from the fields of regional sustainable economic development and natural protection. Among the three function items, the production function refers to the material supplies for human activities, including agricultural production function, along with the second and third industrial production function; the living function signifies dwelling, availability of employment and education opportunities, guarantee of transportation and social security, and leisure entertainment among other human factors; the ecology function, playing a foundation role of an ecosystem for human production and livelihood, refers to a series of ecosystem services [19–22]. Obviously, the production and living function of land-use space are the principle causes for regional ecological risk, whereas the ecology function of land-use space plays a receptor and absorption role of regional ecological risk. Currently, these three function indexes have been broadly used in the expression of pattern and evolution path of regional or urban production, living, and ecology function spaces, and the subsequent characterization of the coordination level of regional/urban development with respect to human disturbance [17–21, 23]. This provides us a viewpoint of land use function to recognize spatial differences of human activities and ecological capitals based on land use and other related data. However, there is few related research using this land use function system for regional ecological risk analysis and assessment purposes.

**Fig 1:**
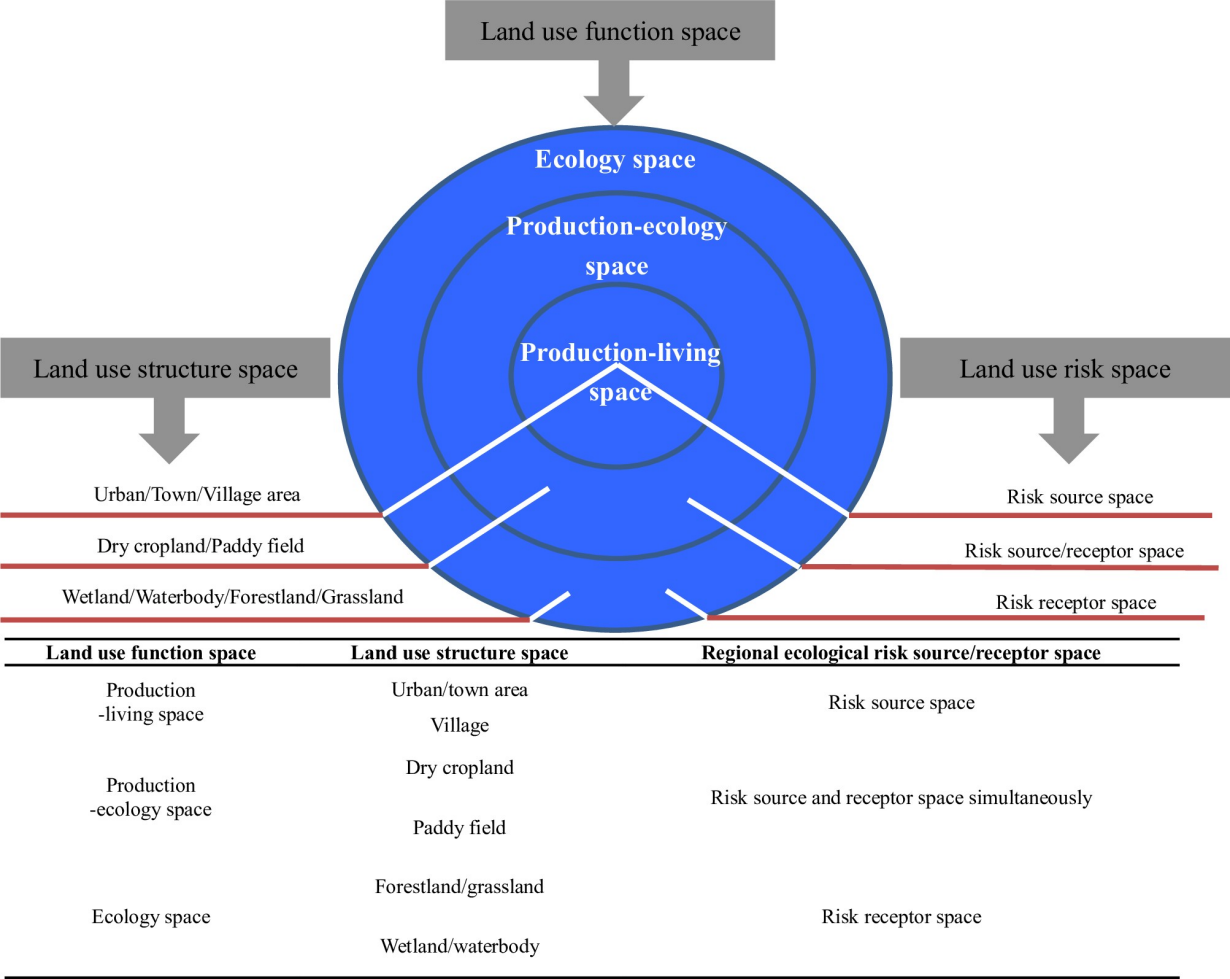
Classification system of regional land use structure, function and risk spaces in this study.

Moreover, on a regional scale, the main function of urban/town/village area (construction land) is production and living, the main function of cropland is production and ecology, whereas the main function of the rest land-use space (forestland, grassland, wetland, and waterbody) is ecology [17, 18, 22, 24, 25]. Thus, the urban/town/village area can be deemed as regional production-living space, cropland can be deemed as regional production-ecology space, and the rest of the land-use space can be referred to as regional ecology space [24, 25]. This reclassification system of regional function space is particularly useful for illustrating the spatial division pattern of human-caused ecological risk source-receptor space regionally based on land use/land cover data. For example, the urban/town/village and cropland areas, with their prominent production and living function, are a source space of regional ecological risk, whereas the ecological land space, including cropland which possesses a certain extent of ecology function (ecosystem services), can be deemed as the receptor space of regional ecological risk. Among the land-use space, urban/town/village construction land is the prime agglomeration area (central place) of regional human activities [26]; therefore, it may be recognized as dominating source space of regional ecological risk [4], whereas the ecological land space (such as forestland, grassland, wetland, and waterbody) can be deemed as main receptor space of regional ecological risk owing to their high ecological capital and low governance level [4]. Additionally, cropland has both significant production and ecology function [20], and thus, it can be regarded as a source and receptor space simultaneously within RERA research. Therefore, land-use space can be characterized by its structure, function, and risk division features as shown in Fig 1. Besides, this figure can also help us understand that why so many researchers [2, 4, 5, 13, 14] consider construction land (urban/town/village area) and cropland, especially construction land, as human disturbance sources for interested ecosystem and its protection in related studies.

Therefore, in this study, based on the land-use function/risk space division scheme (Fig 1), three function indexes of regional land-use space, i.e., production function index, living function index, and ecology function index, were proposed and produced using land use data within the human-caused RERA framework. Among the three indexes, the former two are source indicators of regional ecological risk with respect to human disturbance, whereas the latter is a receptor indicator of regional ecological risk. Besides, as a substitute of artificial assignment method based on land use data [17, 18], the Defense Meteorological Program Operational Line-Scan System (DMSP/OLS) nighttime light intensity data, which matches well the distribution of regional urban/town land [4], were used to produce a regional living function index in the proposed study. Incorporated with eco-environmental vulnerability features [4], the RERA index system was finally established. The Poyang Lake Eco-economic Zone (PLEZ), whose balance between eco-environmental protection and socio-economic development has attracted attention on a national level [27], was selected as the case study area to examine the effectiveness of the proposed RERA model. This study aims to identify, assess, and manage regional ecological risk with respect to human disturbance in accordance with land use risk space division, and thus, reduce its severity level and promote regional sustainable development capacity finally.

## 2. Materials and methods

### 2.1. Study area

**Fig 2:**
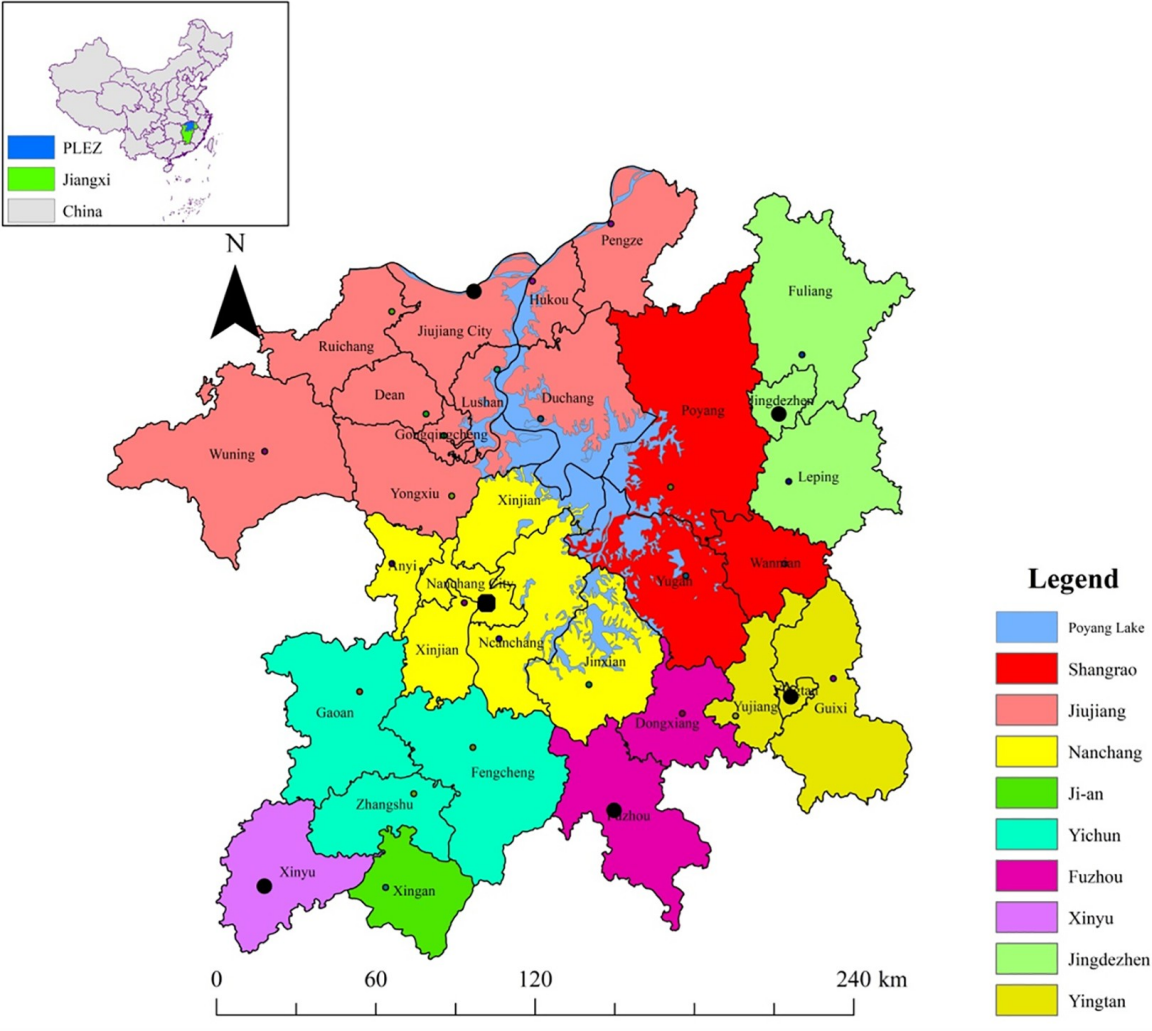
Location and administration division of the PLEZ.

The PLEZ (28°30’N–30°06’N, 114°29’E–117°25’E) is located in the northern part of the Jiangxi Province, China (Fig 2). The region covers approximately 51,200 km^2^ [16]. In administration division terms, the region consists of 6 cities and 24 additional county units. Cities include Nanchang (the capital of the Jiangxi Province), Jiujiang, Jingdezhen, Yingtan, Fuzhou, and Xinyu. Poyang Lake, the largest freshwater lake in China, is in the region’ s central region and receives most of its water from five major rivers, namely the Gan River, Fu River, Xin River, Xiu River, and Rao River in the south, and empties into the Yangtze River in the north. One distinguishing feature of the Poyang Lake is its dramatic water level fluctuation within a year, which results in a remarkable lake size change from approximately 3000 km^2^ in summer (wet season) to <500 km^2^ in winter (dry season) [28]. This significant seasonality and inter-annual water level fluctuation make the lake an important wetland habitat in winter for hundreds of thousands of migratory birds when the water level decline and some previous lake waterbodies become wetlands. In addition, most of the region, especially the heart zone, is a flat lacustrine plain, with mountainous areas near the region’s border (S1 Fig). The dominant land-use types are dry cropland, paddy fields, forestland, grassland, construction land, waterbody, and wetland (S2 Fig). The climate in this region is subtropical humid climate, with an annual average temperature of 16–18°C and an annual average rainfall of 1600 mm [16]. The population of the region is approximately 20.53 million [29], with a gross domestic product of 1223.65 billion yuan in 2019 [30].

### 2.2. Data source

#### 2.2.1 DMSP/OLS nighttime light data

The regional living function index was produced using the DMSP-OLS nighttime light intensity data. This dataset, obtained at night by the OLS sensor of the DMSP satellite, was frequently used to record nighttime light intensity worldwide. Human living activities are concentrated in multi-level central places, such as city/town areas and villages [4, 17, 18, 27]. The DMSP/OLS nighttime light data can be used to efficiently identify the boundaries of urban/town areas [31], and this point has been confirmed by the urban/town spatial expansion research in PLEZ by Zhong et al. [27]. Therefore, the DMSP/OLS nighttime light data were used to represent the scale of human living space characterized as urban/town area in this study. This approach was used as a surrogate for the artificial assignment approach in accordance with land-use types [17, 18]. From this viewpoint, the higher the nighttime light value, the stronger the living function of that point, and vice versa. The DMSP/OLS nighttime light intensity data (2010) were acquired from the Resource and Environment Data Cloud Platform, Institute of Geographic Sciences and Natural Resources Research, Chinese Academy of Sciences (http://www.resdc.cn/, accessed July 2020) [32].

#### 2.2.2 Land-use data

In this study, the raster format land-use data (2015) with 1 km resolution, interpreted from Landsat 8 images by manual visual interpretation approach, were used to produce a regional normalized production function index and regional ecology function index based on land-use types. In accordance with the research purpose, regional land use space was divided into 8 types totally, i.e., paddy field, dry cropland, forestland, grassland, construction land, waterbody, wetland, and unused land (S2 Fig). Through an artificial assignment approach, the values for the production and ecology functions were obtained [4, 18]. The artificial assignment protocols have been reported by Cui et al. [17], Liao et al. [18], Zhuang et al. [33], Tu et al. [34], Zhao et al. [35], and Liu et al. [36]. The 2015 land-use data were obtained from the Resource and Environment Data Cloud Platform, Institute of Geographic Sciences and Natural Resources Research, Chinese Academy of Sciences (http://www.resdc.cn/, accessed July 2020) [32].

#### 2.2.3 Soil data

The soil data with 1 km resolution were used to produce the regional soil erodibility index, a sub-indicator of regional eco-environmental vulnerability, in accordance with different soil types [4]. This dataset was digitized from 1:1000000 national soil distribution map of People’s Republic of China (1995). In addition, using an artificial assignment approach, the soil erodibility value for multiple soil types was determined. The artificial assignment standard in this term is provided by Qi et al. [37]. The soil data were obtained from the Resource and Environment Data Cloud Platform, Institute of Geographic Sciences and Natural Resources Research, Chinese Academy of Sciences (http://www.resdc.cn/, accessed July 2020) [32].

#### 2.2.4 Slope/NDVI/drying index data

In addition to soil erodibility (describing the regional vulnerability feature in terms of soil), the other three indexes were also included to comprehensively characterize the regional eco-environmental vulnerability feature, such as the slope grade indicator (expressing the regional vulnerability feature in regards to topography), vegetation coverage vulnerability indicator (expressed in Normalized Difference Vegetation Index (NDVI) data, manifesting the regional vulnerability feature in regards to vegetation coverage), and drying index (characterizing the regional vulnerability feature in aspects of climate) of the region [3, 4]. These three indexes were produced using regional slope data with 90 m resolution, NDVI data with 1 km resolution, and dryness degree data with 500 m resolution, respectively. All these data were resampled to 1 km resolution to continue the following processing steps in this research. The slope data were acquired from China’s Geospatial Data Cloud (http://www.gscloud.cn/, accessed July 2020) [38]. The other two datasets were obtained from the Resource and Environment Data Cloud Platform, Institute of Geographic Sciences and Natural Resources Research, Chinese Academy of Sciences (http://www.resdc.cn/, accessed July 2020) [32].

The data processing platform was ArcGIS 10.2 version (ESRI, USA), and the Albers Conic Equal Area Projection System was used as the mapping standard in this study. In addition, to promote the advancement of RERA within the ArcGIS platform, all the original datasets were first resampled to 1 × 1 km grid cells before they were processed further.

### 2.3. RERA framework for the study area

#### 2.3.1 Integrated risk assessment model

Integrated regional ecological risk was calculated as follows [3, 4]:

R=f(H)×g(E)×h(V), (1)

where *R* represents the final ecological risk value, which reflects the integrated risk level of a region under consideration of ecological risk source intensity, ecological assets value, and eco-environmental vulnerability; *H* represents the human-caused risk source intensity produced by weighted overlaying of production and living function indexes; *E* represents the regional ecological capital (ecology function index); *V* represents the regional eco-environmental vulnerability; and *f*, *g*, *and h* represent different calculation functions for variables *H*, *E*, and *V*, respectively. In accordance with the principles of the RRM model [9], all these three indicators (*H*, *E*, and *V*) were normalized (non-dimensionalization) first using the “Natural Break” function (Jenks) in ArcGIS 10.2 before integrating them to produce the final ecological risk value (*R*). Owing to its merits in identifying the break points between each category naturally and then maximizing the differences between them, the “Natural Break” function was usually employed in the classification procedure within RERA research [3, 4, 12, 13, 15], and therefore, was adopted as well in this study for classifying related indexes into four grades. As for the normalized values of *H*, *E*, and *V*, 1 denotes the lowest risk source intensity, ecological capital value, and eco-environmental vulnerability, whereas 4 denotes the highest. Finally, the integrated ecological risk (*R*) was partitioned into 4 grades with 4 indicating the highest final risk grade and 1 indicating the lowest.

**Fig 3:**
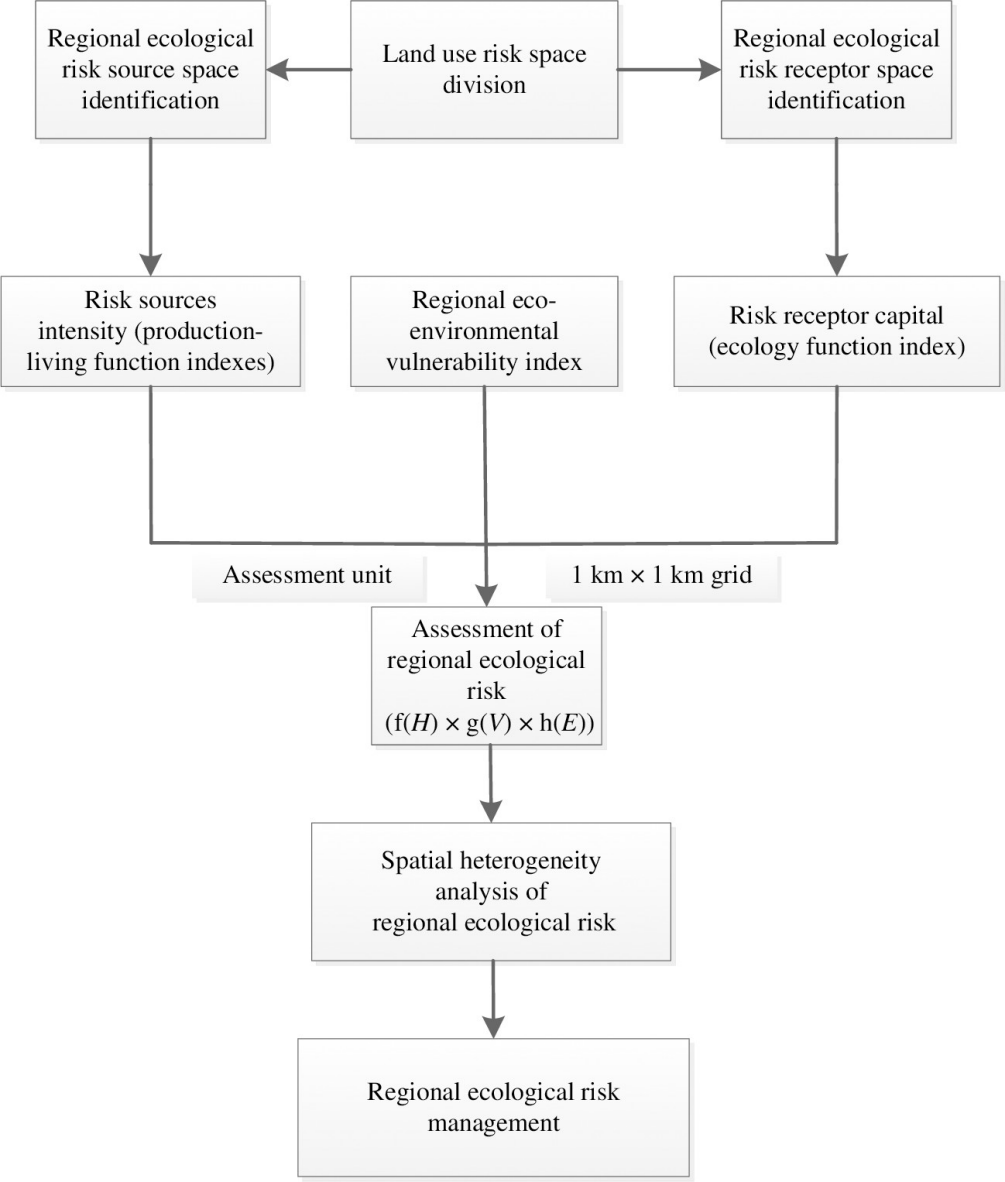
Framework of RERA in the PLEZ.

The framework of RERA in the PLEZ is shown in Fig 3.

#### 2.3.2 Risk source intensity assessment model

The regional ecological risk source intensity was calculated as follows [3, 4]:

$$H=\sum_{m=1}^{t}\left(w_{m}f_{m}\right),$$ (2)

where *H* is the ecological risk source intensity, *t* is the number of risk source factors, *m* is a discrete integer satisfying 0 < *m* < *t* + 1, *f*_*m*_ is the normalized risk source factor (i.e., production function index and living function index, respectively, in this study) for *m*, *w*_*m*_ is the weight of *f*_*m*_ (0.5 for both production and living function indexes in this study), and m is a discrete integer. There are 4 grades for normalized values of *H* and *f*_*m*_, with 1, 2, 3, and 4 indicating low, medium, high, and very high values, respectively. The “Natural Break” function in ArcGIS 10.2 was used to realize this normalization purpose.

According to the artificial land production function assignment and land-use intensity research [17, 18, 33], where land use intensity and production function value increase from ecological land (forestland/grassland, wetland/waterbody) to cropland, and then to construction, the values of production function index of construction land, cropland (including paddy field and dry cropland), forestland, grassland, wetland, waterbody, and unused land were assigned the values of 4, 3, 1, 1, 0, 2, and 0, respectively, in this study. Owing to its water transportation function, the normalized production function value of the waterbody was assigned 2 in this study, in contrast with wetland (0) and forestland/grassland (1). The higher the normalized production index value, the greater the production function of that point, and vice versa.

The DMSP/OLS nighttime light data were divided into five grades using “Natural Break” function in ArcGIS 10.2, among which the non-zero nighttime light value area was assigned 4, 3, 2, 1, in accordance with their light values, whereas areas with zero nighttime light value were assigned 0. The higher the nighttime light value, the greater the living function of that point, and vice versa. The non-zero nighttime light value area comprehensively denotes the scale of the regional human living function space [4, 27, 31].

#### 2.3.3 Ecological capital assessment model

In this study, regional ecological capital, i.e., the regional ecology function index, was produced by adding the value of each kind of ecosystem service per unit area based on land-use types [3, 4, 18]. These ecosystem services can be divided into 9 types according to Xie et al. [39]: gas regulation, climate regulation, water conservation, soil formation and protection, waste treatment, biodiversity conservation, food production, raw material, and entertainment culture. In this study, construction land and cropland areas have a certain degree of ecosystem services resulting from the multi-functionality of land [34–36]. Therefore, these two land-use types were given ecological capital values in this study. The comprehensive ecosystem service value per unit area of all land-use types was generated by overlaying the values of single ecosystem service one by one [39, 40].

Subsequently, the integrated ecosystem service value per unit area of all ecosystems was normalized to 1, 2, 3, and 4, respectively, by the equal distance division approach [4, 14]. This classification method was adopted because it can emphasize on the direct comparison of numerical values of ecosystem service index, which was an artificial assignment product of land use data, in contrast with those natural numerical products, such as the living function index. The higher the integrated ecosystem service value, the stronger the ecology function of that point, and vice versa. According to the related studies [34–36], grade 1 refers to dry cropland, paddy field, unused land, and construction land together, grade 2 refers to grassland area, grade 3 refers to forestland area, and grade 4 refers to wetland and waterbody (mainly Poyang Lake).

#### 2.3.4 Environmental vulnerability assessment model

The regional eco-environmental vulnerability index was calculated as follows [3, 4]:

$$V=\sum_{n=1}^{l}\left(w_{n}f_{n}\right),$$ (3)

where *V* represents the final eco-environmental vulnerability, *f*_*n*_ represents the normalized vulnerability factors in this study (i.e., 4 grades with 1, 2, 3, and 4 denoting low, medium, high, and very high vulnerability levels, respectively), *w*_*n*_ represents the weights of *f*_*n*_, *l* represents the number of eco-environmental vulnerability factors, and 0 < *n* < *l* + 1, where n is a discrete integer.

Eco-environmental vulnerability can influence the damage degree of ecosystems stemming from human activities [3, 4]. Therefore, the corresponding regional eco-environmental vulnerability indexes were also included in this study. To comprehensively express the spatial heterogeneity of eco-environmental vulnerability (*V*), four factors were considered in this study [4], including slope (denoting regional landform feature), NDVI (representing regional vegetation cover feature), drying index (characterizing regional climate attribute), and soil erodibility (manifesting regional soil feature). Among these, the values of last index were assigned artificially according to soil types and the artificial assignment standard in this term is provided by Qi et al. [37]. Then, these values were standardized into four grades before processing further using the equal distance division approach [4, 14]. This method was employed because I want to highlight the direct comparison of numerical values of this artificial assignment index here. On the other hand, the former three indexes were normalized into four grades through “Natural Break” function, which was preferred in related RERA research [3, 4, 12, 13, 15], based on corresponding datasets.

In general, the degree of regional vulnerability level increases with higher slope, drying index, soil erodibility values, and lower vegetation coverage, and vice versa [3, 4]. Therefore, the division scheme of regional vulnerability grades in terms of these factors was realized as shown in Table 1. The weights of slope, NDVI, drying index, and soil erodibility were 0.3, 0.3, 0.2, and 0.2, respectively, in accordance with their relative importance in eco-environmental vulnerability evaluation [3, 4].

**Table 1:** Indexes used for eco-environmental vulnerability assessment in the PLEZ.

Factors	Grades Assigned				Weight
	1	2	3	4	
**Slope (°)**	< 4.36	4.36–11.61	11.61–21.05	> 21.05	0.3
**Vegetation Coverage (%)**	> 78.35	61.47–78.35	35.24–61.47	< 35.24	0.3
**Drying Index**	< 560	560–652	652–720	> 720	0.2
**Soil Erodibility (K value)**	< 0.13	0.13–0.21	0.21–0.30	> 0.30	0.2

## 3. Results

### 3.1. Ecological risk source intensity

**Fig 4:**
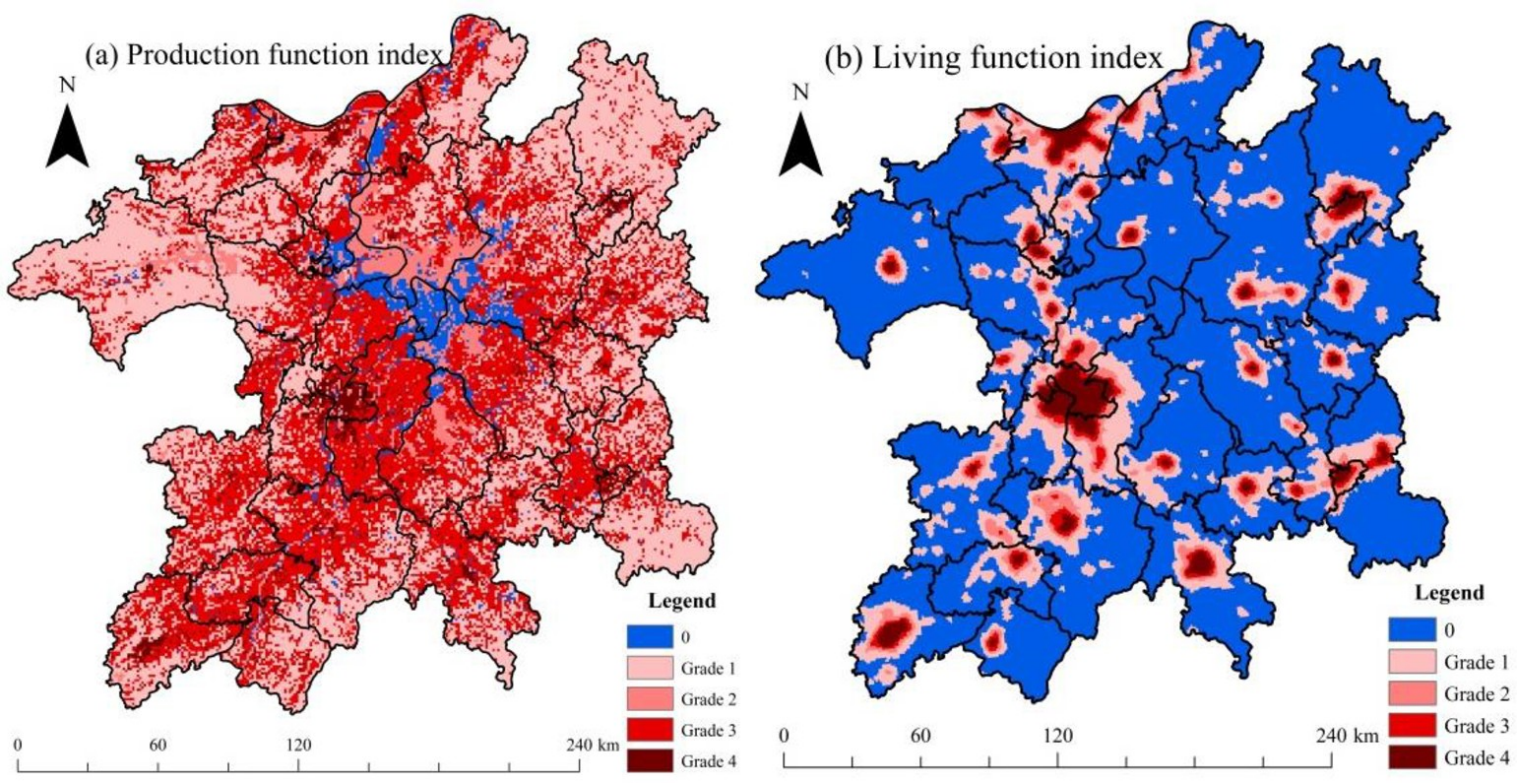
Intensity grades of regional production and living function index in the PLEZ.

The intensity distributions of regional production function and living function index are shown in Fig 4. These intensity distributions of the two indexes were in accordance with regional land-use function/risk space division scheme in Fig 1. From Fig 4(A), we can see that the high production functions (grades 3 and 4, denoting construction land and cropland area, respectively) were mainly distributed in the Gan-Fu River fluvial plain to the southwest of Poyang Lake and lacustrine plain area to the southeast of the lake. City downtown areas, such as Jiujiang, Jingdezhen, and Yingtan, were also characterized in the high production function grades. The zero-production function region was the winter wetlands along Poyang Lake. The grade 2 production function region was mainly the winter waterbody area of the lake, whereas grade 1 production function region (forestland/grassland) was mainly distributed in the four corners of the zone.

From Fig 4(B), in comparison with regional land use situation (S2 Fig), we can find that the non-zero area of the DMSP/OLS nighttime light data matched well with the distribution of regional urban/town area, such as the cities of Nanchang, Jiujiang, Fuzhou, Jingdezhen, and Xinyu. Moreover, the county seat scope of the zone was also evident with relatively smaller nighttime light areas, such as Duchang, Wanning, and Gongqingcheng, among others.

**Fig 5:**
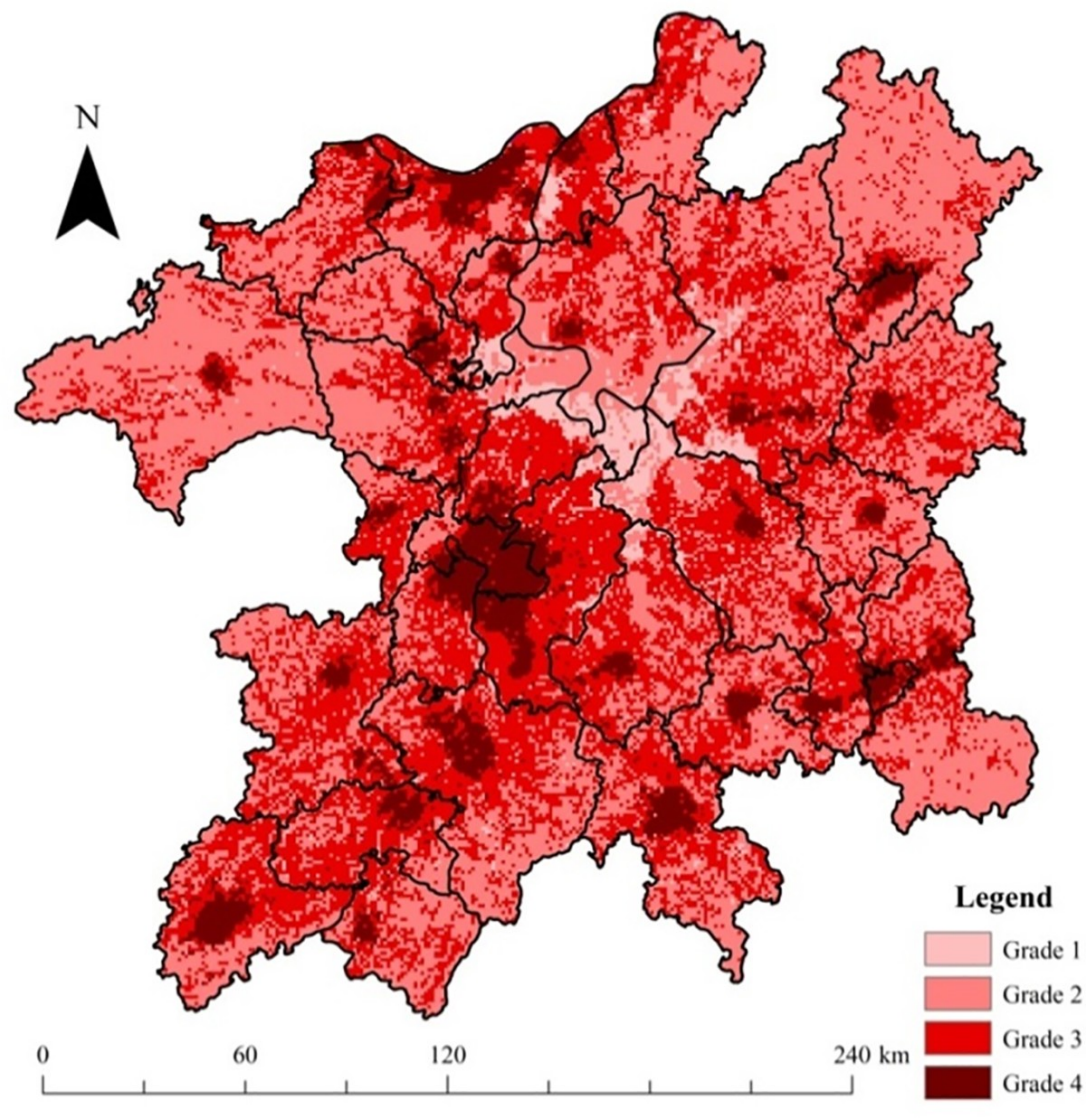
Integrated intensity grades of regional ecological risk source in the PLEZ.

Based on Formula 2, the integrated risk source intensity of the PLEZ was calculated, as shown in Fig 5. From Fig 5, the very high-risk source intensity area (grade 4) mainly reflected the scope of urban/town construction land, whereas the high-risk source intensity (grade 3) displayed the distribution of croplands. The forestlands had medium integrated risk source intensity levels (such as the four corner areas within the PLEZ), as did the winter waterbody of Poyang Lake. However, the winter wetlands of Poyang Lake had the lowest integrated risk source intensity level (grade 1) owing to its lowest human disturbance level. From the viewpoint of proportion, the low, medium, high, and very high grades of ecological risk source intensity accounted for 3.64%, 48.37%, 39.71%, and 8.28% of the region, respectively. The area of the middle risk source intensity (grades 2 and 3) is relatively larger.

### 3.2. Ecology function index distribution

**Fig 6:**
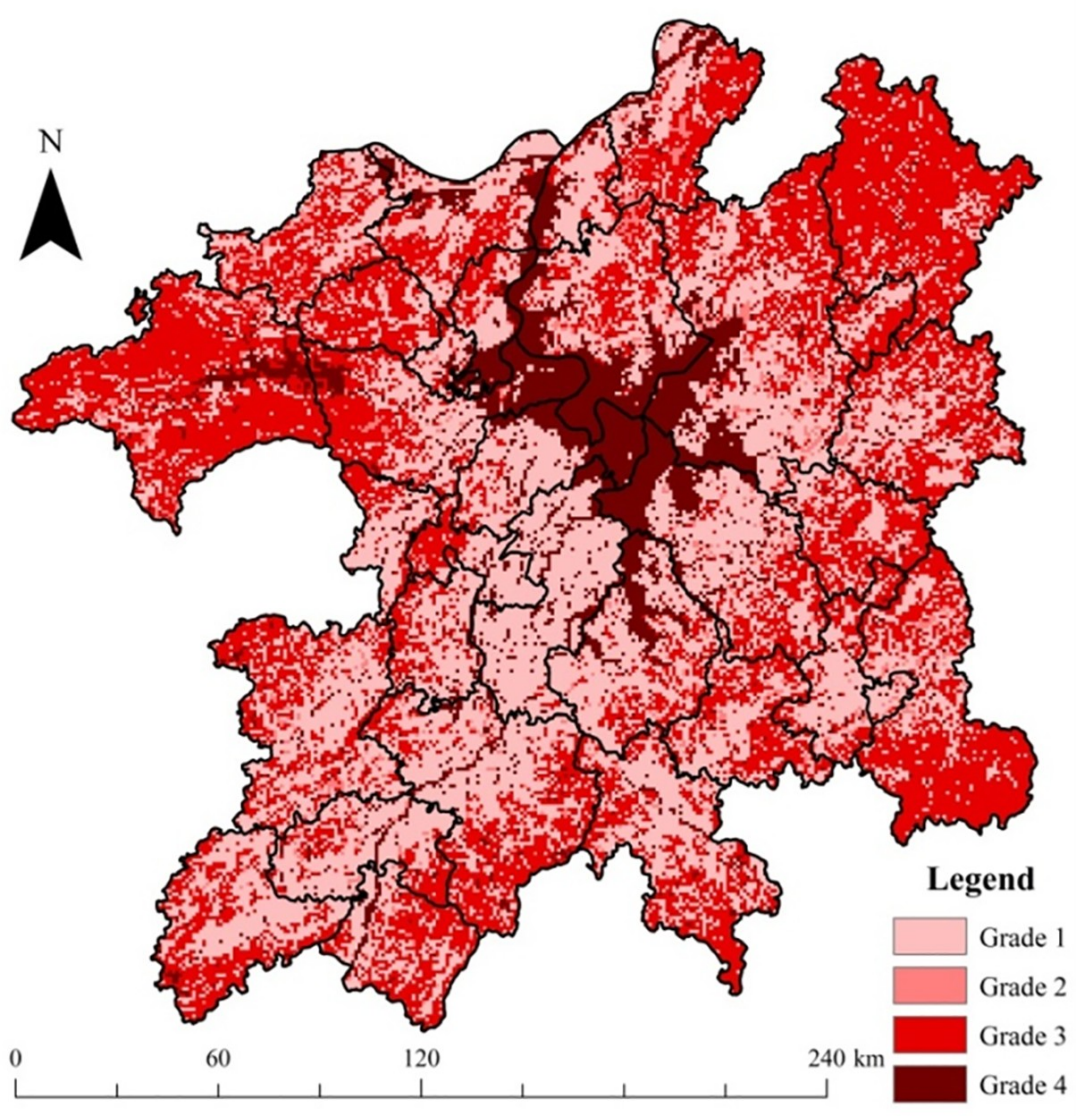
Grades of ecology function index in the PLEZ.

The grades of regional ecology function indexes are shown in Fig 6. These grades of the index were in line with regional land-use function/risk space division scheme in Fig 1. From Fig 6, grade 4 (wetlands/waterbody), grade 3 (forestland), and grade 2 (grassland) represented the scope of regional ecological land space, whereas the grade 1 area represented the distribution of dry cropland, paddy fields, construction land, and unused land of the region. From the viewpoint of the proportion, the low, medium, high, and very high grades of ecological capital accounted for 43.48%, 3.77%, 40.55%, and 12.20% of the whole region, respectively. Most of the region is covered by low (grade 1) and high (grade 3) grades of ecology function. However, the wetlands/waterbodies, with the highest ecosystem service provision function, still covered a considerable area of the zone (12.20%).

### 3.3. Eco-environmental vulnerability distribution

**Fig 7:**
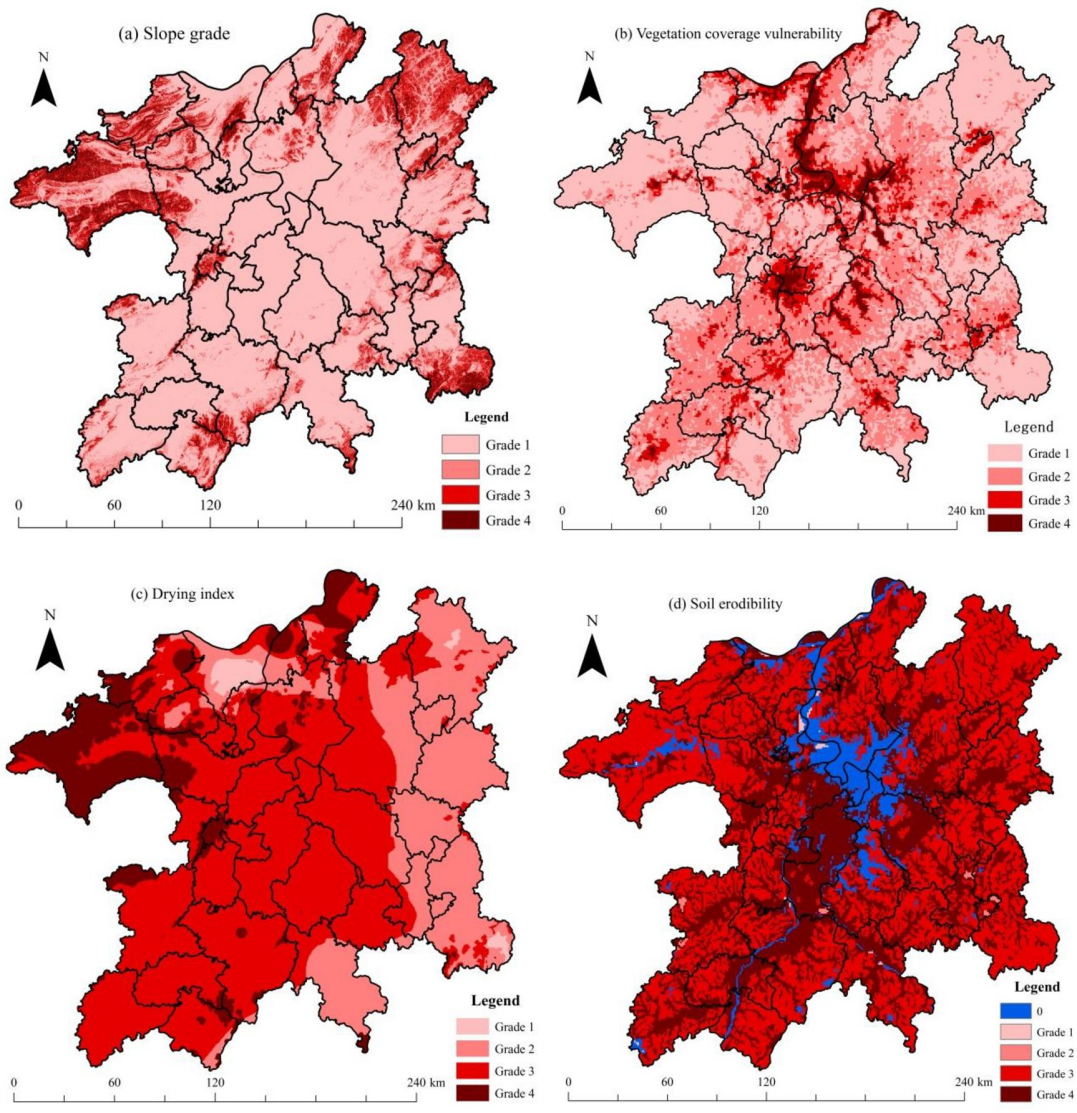
Grades of vulnerability factors in the PLEZ in aspects of (a) slope, (b) NDVI, (c) drying index, and (d) soil erodibility.

The distributions of four sub-vulnerability indexes in the region are shown in Fig 7. From Fig 7(A), we can observe that the majority of the study area was covered by low slope vulnerability (grade 1), which indicated that most of the PLEZ was relatively flat. The large slope area (grades 3 and 4) was mainly distributed in the northeast, northwest, and southeast corners of the region. From Fig 7(B), the high and very high grades of vegetation coverage vulnerability (grades 3 and 4) were evident in the distribution of winter waterbodies of Poyang Lake and urban construction land, such as the Nanchang City area. In Fig 7(C), most of the zone was characterized by high and very high drying index grades, while only the eastern part of the region was characterized by the medium-drying index grade (grade 2). From Fig 7(D), we can observe that the soil erodibility level of the region was high, with almost the entire study area characterized by high and very high soil erodibility potential (grade 3 and grade 4).

**Fig 8:**
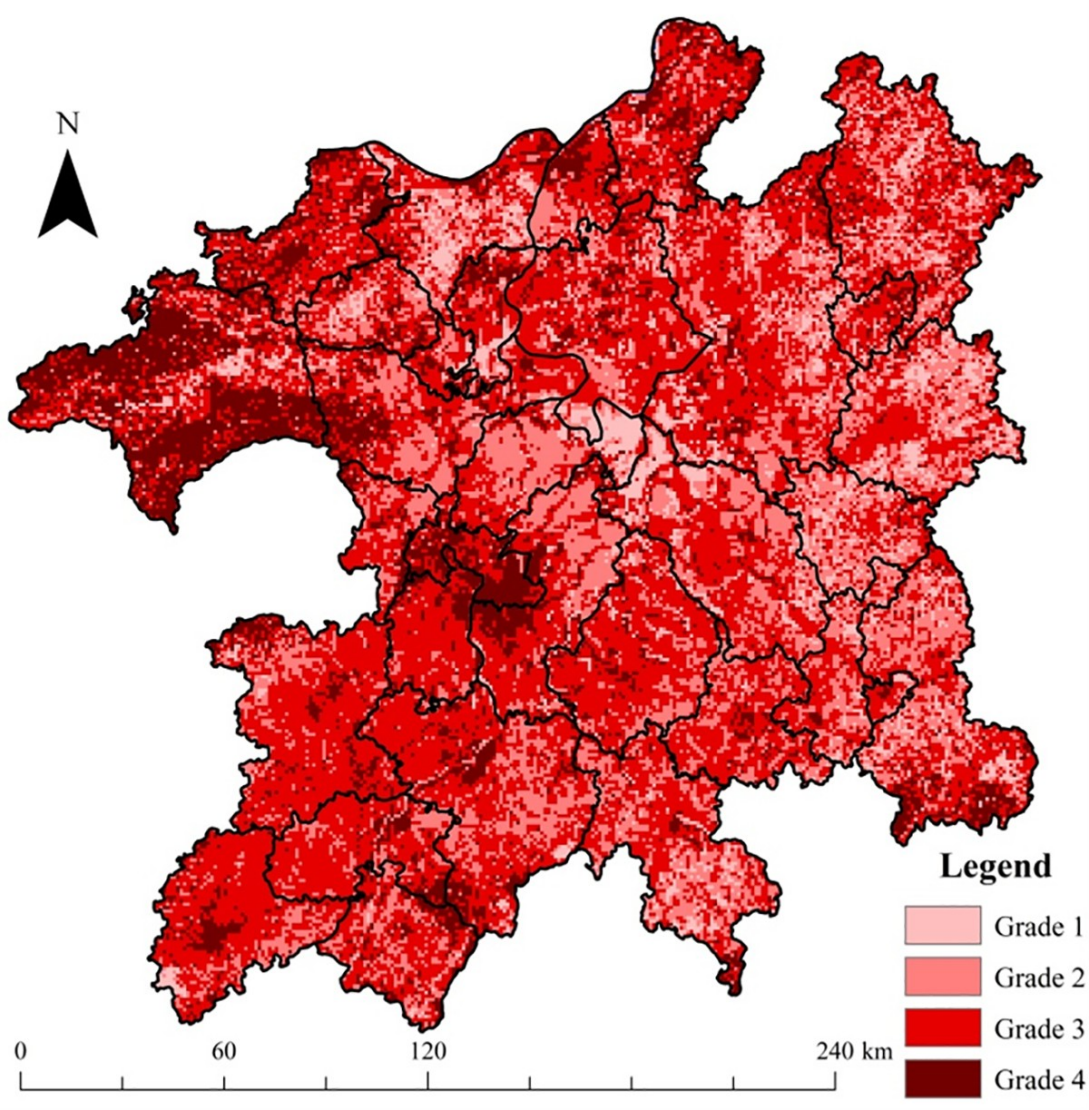
Grades of integrated eco-environmental vulnerability in the PLEZ.

Based on Formula 3, the final integrated eco-environmental vulnerability of the PLEZ was calculated (Fig 8). In general, most of the zones were located in the scope of high and very high eco-environmental vulnerability levels (grades 3 and 4). The western part of the zone had a higher eco-environmental vulnerability level overall than that of the eastern part. The urban construction land area, such as the Nanchang City region, with a low NDVI value, was in the very high vulnerability grade (grade 4), as was the forestland in the northwest corner of the zone. From the viewpoint of proportion, the low, medium, high, and very high grades of vulnerability accounted for 6.92%, 26.91%, 50.06%, and 16.11% of the region, respectively.

### 3.4. Integrated ecological risk degree in the PLEZ

**Fig 9:**
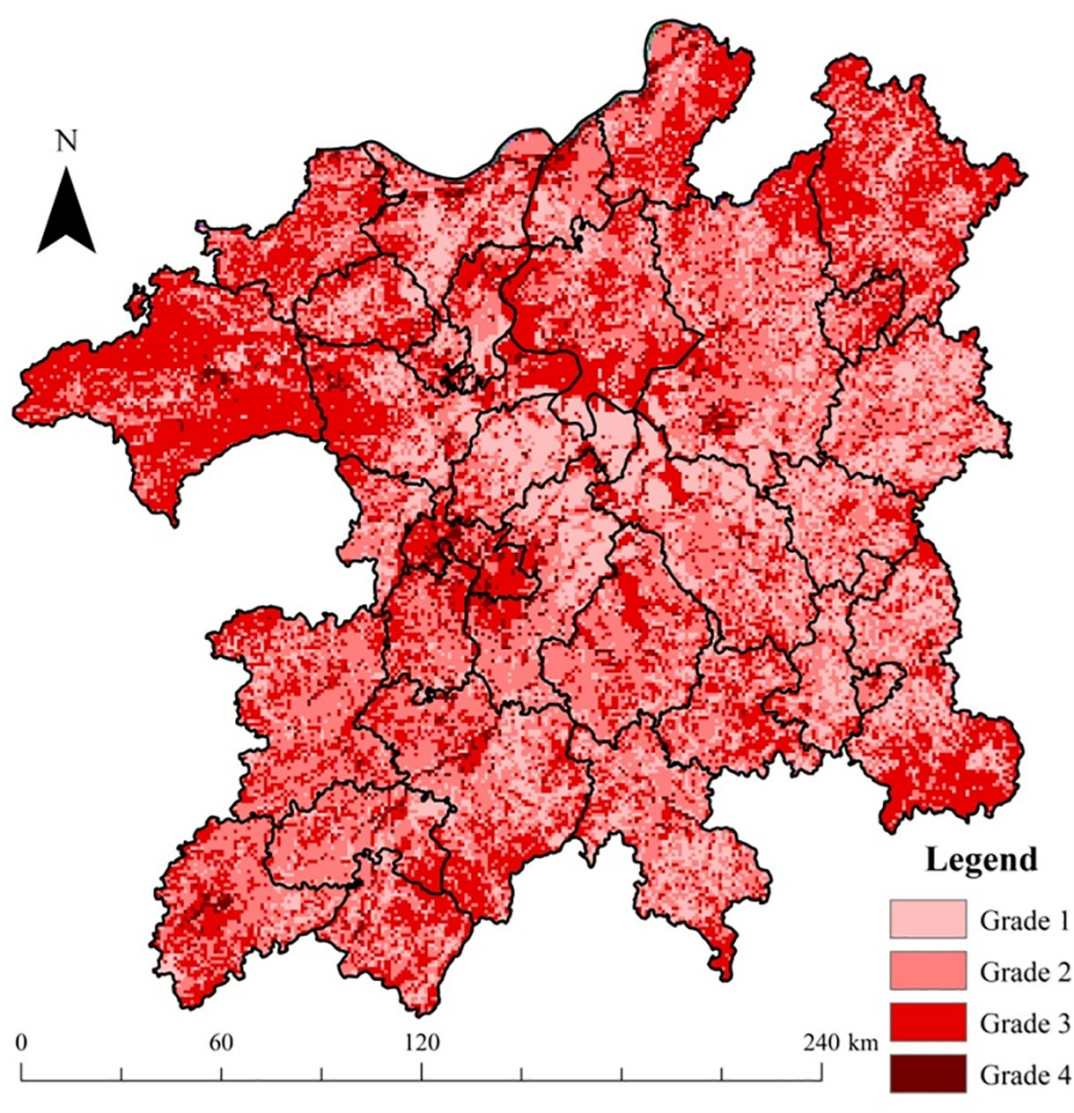
Distribution of final risk grades in the PLEZ.

Based on Formula 1, regional integrated risk grades (considering the ecological risk source intensity, ecological capital value, and eco-environmental vulnerability comprehensively within a region) were calculated and are shown in Fig 9. In Fig 9, the distribution of very high final risk level (grade 4) was significantly limited, i.e., only 2.94% of the whole PLEZ. The winter waterbody of Poyang Lake was mainly of high risk (grade 3), as was the Nanchang City construction land area. In contrast, the final risk level of winter wetlands was relatively low (grade 1). In addition, the forestland within the boundary region of the zone was also almost in the high-risk grade (grade 3), while the risk level of croplands was mainly located in low grades (grades 1 and 2). Owing to the highest human disturbance (manifested in both production and living function indexes) and eco-environmental vulnerability level, the urban/town area, such as Nanchang City, had the highest final risk grade. The difference in the final risk level between winter waterbodies and wetlands is mainly caused by their different production function indexes (2 for winter water body of Poyang Lake and 0 for wetlands). The higher NDVI vulnerability for winter waterbodies also contributed to this higher final risk level. The low, medium, high, and very high-risk grades accounted for 21.22%, 39.53%, 36.31%, and 2.94% of the whole region, respectively.

## 4. Discussion

### 4.1. Territorial spatial planning/governance for different land-use function/risk spaces within the PLEZ

When it comes to the RERM discussion, territorial spatial planning/governance for different land-use function/risk spaces will be very helpful. In this research, based on the land-use situation of the PLEZ (S2 Fig), regional land-use function/risk space can be divided into four types, i.e., production-living space (urban/town/village development zone-regional ecological risk source space), production-ecology space (agricultural production zone and regional ecological risk source/receptor space), forestland ecology space (regional ecological risk receptor space), and wetland-waterbody ecology land space (regional ecological risk receptor space), to better control the severity level of regional ecological risk caused by human activities, thereby realizing regional sustainable development goals.

#### 4.1.1 Production-living space

The results (Fig 9) show that urban/town areas, such as Nanchang, Jiujiang, and Xinyu Cities, had the highest final risk grade. This situation was principally caused by the high human disturbance level there, including both production and living human activities. Therefore, for this regional ecological risk source space, controlling and reducing ecological risk source intensity, such as defining boundaries for spatial urban/town/village growth as a redline strategy is necessary. In general, the expansion of urban/town area suggests the enlargement of regional production-living function space and reinforcement of human activity intensity of the region. Correspondingly, the severity of regional ecological risk stemming from human activities will also be elevated. Some research has found that the best method to mitigate the degradation trend of regional ecosystem services provision status is to limit the spatial expansion of urban/town land space [5]. Therefore, it is necessary to strictly restrict the unreasonable expansion of city/town construction land area to control the increase of ecological risk level in the region. Besides, within this land use risk source space, promoting production efficiency by acquiring new techniques, adopting cleaner production, industrial ecology, and circular economy principles [41] for improving the natural resource utilization rate, and advocating moderate consumption, are all useful approaches for controlling and reducing regional ecological risk level purposes.

#### 4.1.2 Production-ecology space

As shown in Fig 9, the risk level of croplands was mainly located in the low grades (grades 1 and 2). Nevertheless, further management/precaution approaches are still required. For this regional ecological risk source/receptor space, for example, the tradeoff between production and ecology function of agricultural land space will be helpful [20]. In farming practice, one tends to emphasize on the agricultural production capacity, but neglect the provision of ecosystem services of croplands [4], such as soil and water conservation functions, nitrogen and phosphorous nutrient maintenance functions, and other similar services. In practice, the overuse of fertilizer and pesticide to compensate for the loss of cropland fertility is pervasive. It possibly results in heavily polluted downstream waterbody and wetland areas [42, 43] through soil erosion and non-point source pollution pathways [44, 45]. Thus, tradeoff strategies [4, 20] between the production and ecology function of cropland, such as rational fertilization, contour tillage, and returning farmland to forestland in steep slope areas, are crucial. In other words, more attention should be paid toward the balance between the two sub-functions of cropland, rather than solely highlighting its single production function. These practices will be helpful for controlling soil erosion and nutrient loss issues in this source landscape area and reducing the ecological risk level of downstream wetlands and waterbody areas [4].

#### 4.1.3 Forestland ecology space

In Fig 9, the forestland within the boundary region of the zone was nearly classified in the high-risk grade (grade 3). Therefore, greater attention should be paid toward forestland ecology space protection. From this viewpoint, coordination and optimization of economic exploitation activities within forestland ecology space are promising for controlling and reducing regional ecological risk. As one of the main ecology function spaces within the PLEZ, forestlands can be exploited to a limited increase in biological resources, such as log, fruit, tea, oil, bamboo, and wild vegetable, among other similar resources. Unreasonable natural resource utilization can destroy the health status and ecosystem services provision function of this ecosystem, resulting in soil erosion and nutrient loss issues within their boundaries. Thus, more attention should be paid toward the balancing of the exploitation of natural resources and effective ecological protection within forestland ecology space, which, in turn, will contribute in controlling the soil erosion and nutrient loss issues in this regional ecological risk receptor space.

Moreover, geomorphic factors have important effects on the severity of ecological damage caused by human activities, such as soil erosion and nitrogen and phosphorus nutrient loss. Thus, special attention should be paid on fragile and sensitive forestland areas, especially steep slope forest regions. Related approaches include setting up a restricted development zone or forestland nature reserves [46] within this type of land use function space, to avoid the harmful ecological consequences. From this viewpoint, new economic exploitation activities must be conducted in accordance with the ecological suitability of the forestland region.

#### 4.1.4 Waterbody/Wetland ecology space

From Fig 9, we can observe that the winter waterbody of Poyang Lake was mostly characterized by the high-risk grade (grade 3), while the final risk level of winter wetlands was relatively low (grade 1). Apart from the higher NDVI vulnerability for winter waterbody than that of wetlands, this can be attributed to the fact that the production function index of winter waterbody (2) was larger than that of winter wetlands (0). Therefore, for the winter waterbody area of Poyang Lake, controlling its production utilization intensity, especially navigation activities, can significantly promote ecological risk reduction.

Owing to its significant intra-year water-level change, a part of the Poyang Lake waterbody will be exposed to air when the drawdown period starts in the fall [47]. Therefore, during the drawdown period of the lake, the wetland areas play an important role in supporting the inhabitation of many valuable wintering birds [28], such as the Siberian Crane (*Grus leucogeranus*) [48]. Furthermore, it is necessary to take effective measures to protect these key regional ecological spaces [49]. One of the related approaches is setting up a nature reserve to implement the ecological protection redline strategy of wetlands. To date, two national wetland nature reserves, namely the Poyang Lake National Nature Reserve and Nanjishan National Nature Reserve [50], have already been established in the PLEZ to protect the wetland wintering birds and their environment.

Nature reserve can be employed in practice for wetland protection as a strictly protected area (PA). In some studies, strictly protected areas have been shown to be the most effective way to protect biodiversity and ecological space of target land space [51, 52]. Moreover, the bottom-up protection method, such as community participation, has also been demonstrated as an efficient way to guard the natural space and its biodiversity [53, 54]. Thus, considering these approaches, the zoning of the wetland area can be a feasible spatial governance way to coordinate natural protection and nearby community subsistence demands, such as “core zone-buffer zone-experimental zone” the ring-shape spatial governance division scheme. Through this tradeoff, the efficient protection of these vitally protected areas can be realized. Clearly, the wetland resource utilization by nearby communities in experimental and buffer zones should be supervised and controlled timely, avoiding the appearance of PA downscaling, downsizing, and degazettement (PADDD) events [55–57].

### 4.2. Progress/limitation of the work and further improvements needed

Herein, for the first time, the land use risk space division (regional ecological risk source/receptor space identification) was incorporated into human-caused RERA and RERM research through using production–living–ecology analysis. Because the production and living functions are both active acquisition and then consumption activities of natural resources, these two indicators can be deemed as regionally direct ecological risk sources [4] for multiple ecosystems, i.e., cropland, wetland, waterbody, forestland and grassland. Owing to the conciseness of the proposed RERA model, this method could be referenced widely in the following studies. Additionally, in terms of data acquisition and spatial production–living–ecology function expression, the artificial assignment method was implemented to characterize regional production and ecology function indexes based on land-use data, and its substitute to acquire DMSP/OLS nighttime light data for producing a regional living function index [17, 18]. Because of the convenience with regards to data acquisition and processing, these two datasets have great potential to be adopted in the future. Compared with the proposed RERA framework in this paper, the land use intensity RERA research [14] only emphasizes on the regional production function index essentially, without considering the spatial heterogeneities of regional living function, regional ecological capital, and regional eco-environmental vulnerability level. Moreover, the county-scale administrative units [12] employed in the Poyang-Lake-Region RERA research using production–living–ecology analysis are unable to express the regional production–living function differences within those units, which, by contrast, can be manifested based on land use and DMSP/OLS nighttime light data in this research.

Of course, there still some limitations exist in this research model. For example, besides human activities, climate change [58, 59] and natural hazards, especially flood and drought hazards [60, 61], are all severe challenges faced by the PLEZ; therefore, in the future, these two ecological risk sources should be incorporated into the integrated PLEZ-RERA framework rationally on a regional scale. Moreover, compared with landscape disturbance [15, 16] and land use intensity [14] ecological risk assessment research, the proposed study is a static RERA research, without considering the temporary change traits of regional ecological risk level. The Multi-period contrastive RERA research in PLEZ should be considered in the following related studies. Last but not least, in this research, only the first-level land use types, e.g., forestland, waterbody and construction land (or its surrogate DMSP/OLS data) (S2 Fig), were employed in regional production and living function indexes assignment procedure, without considering the differences between those second-level land use types which were grouped into the same first-level land use type, such as the situation between ecological forest and economic forest, fishpond and natural waterbody for production function value, and urban/town/village area with industrial park for living function value [18]. This treatment method of land use data also happened to those ecosystem service and land use intensity artificial assignment studies [4, 14, 33–36, 39]. Nevertheless, in the future, more accurate land use data, such as that with second-level classification system [17, 18], should be used to better realize regional production-living-ecology function value assignment purpose through identifying the differences in terms of these three functions between second-level land use types. Of course, this elevation of spatial resolution of regional production and living function indexes will undoubtedly increase the workloads and difficulties of RERA result analysis finally.

### 4.3. Policy recommendation for regional development-protection coordination purpose

Related policies recommended for regional development/protection coordination purpose include urban/town development boundary delimitation [5] policy for its expansion governance, industrial ecology policy [62] for industries development within urban/town area, and “ecological redline” (e.g., establishing nature reserves) [50, 63] and/or “Grain for Green” [64] policies for the protection of wetland, lake, forestland, and other important ecosystems regionally. For example, from the viewpoint of risk source intensity reduction, industrial ecology policy can be practiced, embodying cleaner production, low-carbon economy, industrial symbiosis, and circular economy modes [41] for improving the natural resource utilization rate, and then promoting production efficiency. From the viewpoint of ecological capital protection, the useful “ecological redline” policies could be the strictly protected area approach or grass-roots community participation method, which have been proven as effective methods for realizing this purpose [51, 53, 54]. Finally, the “Grain for Green” policy can be adopted through returning the reclaimed farmland back to lake [65], wetland [66, 67], and forestland [68] in zones with high ecological capital level and/or high eco-environmental vulnerability degree [46]. Through all these policies, one should ensure that within the territorial space, “the production land space is used intensively and efficiently, living land space is livable and proper in size, and ecology land space is unspoiled and beautiful.” [18]

As an environment-friendly and resource-saving regional development policy, the ecological economic development mode has been effectively practiced in PLEZ since its establishment in 2009 [27]. Continuously promoting ecological economic development in this zone can help solidify the ecological civilization concept [22] in the people. Consequently, the human behaviors of resource exploitation and utilization along with consumption customs, can be optimized, which, in turn, will undoubtedly facilitate the sustainable development of this ecological economic zone.

## 5. Conclusions

Herein, using the production–living–ecology analysis of land-use function space, we identified the regional ecological risk source/receptor spaces with respect to human disturbance based on land use data. The production-living function space, specifically urban/town/village construction land area, can be classified as a regional ecological risk source space, whereas the ecology function space, i.e., forestland/grassland and wetland/waterbody area, can be grouped into regional ecological risk receptor space. Moreover, owing to its significant production and ecology function, the cropland, namely the production-ecology function space, can be recognized as regional ecological risk source/receptor space simultaneously. Based on this regional ecological risk source/receptor space division scheme, three land use function indexes were proposed for RERA, i.e., production function index, living function index, and ecology function index. Among them, the production and living function indexes can be regarded as regional ecological risk source indicators, whereas the final one can be deemed as a regional ecological risk receptor indicator. Incorporated with the eco-environmental vulnerability index, an RERA framework with respect to human disturbance was established in this study.

The PLEZ-RERA research results indicated that: (1) The DMSP/OLS nighttime light intensity data can characterize the distributions of urban/town area well, and it was rational to employ this dataset to produce the regional living function index. (2) Overall, the forestlands and winter waterbodies of Poyang Lake were in the high-risk grade, as was the Nanchang City construction land area; in contrast, the final risk level of winter wetlands and croplands was relatively low. (3) Owing to the highest human disturbance (including both production and living human activities) and eco-environmental vulnerability level, the urban/town area, such as Nanchang City, had the highest final risk grade. (4) Finally, from the viewpoint of proportion, the low, medium, high, and very high-risk grades accounted for 21.22%, 39.53%, 36.31%, and 2.94% of the region, respectively. Moreover, some RERM measures, such as controlling the irrational expansion of urban/town area, strictly protected area approach and grass-roots community participation for the protection of wetland nature reserves, and the tradeoff between the production and ecology function of cropland, are helpful for promoting regional sustainable development of the PLEZ.

## Supporting information

S1 Fig Digital elevation model (DEM) of the PLEZ. (TIF)

S2 Fig Land use/land cover of the PLEZ. (TIF)
